# Experiences on Role Transition Among Nurse Prescribers

**DOI:** 10.1111/inr.70047

**Published:** 2025-06-13

**Authors:** Jonna Suominen, Leena Salminen, Marja Renholm, Virpi Sulosaari, Heli Virtanen

**Affiliations:** ^1^ University of Turku; Laurea University of Applied Sciences Turku Finland; ^2^ Health Pedagogy at University of Turku, Turku University Hospital Turku Finland; ^3^ Helsinki and Uusimaa Hospital District Helsinki Finland; ^4^ University of Turku; University of Applied Sciences of Turku Turku Finland; ^5^ University of Turku; Turku University Hospital Turku Finland

**Keywords:** competence, inductive content analysis, non‐medical prescribing, nurse prescribing, qualitative research, role transition

## Abstract

**Aim:**

The aim was to describe the experiences of nurse prescribers on role transition during nurse prescriber education and working in a new role.

**Background:**

Nurse prescribing has increased globally, bringing a new importance for nursing. Transition into a new nursing role can be motivating, satisfying, and empowering. However, it can also be stressful and complex. It is important to know how the role transition from a nurse to a nurse prescriber could already be facilitated during studies and the first working years in a new role. As little work has been conducted on this topic, there is a gap in this research area.

**Methods:**

This phenomenological study was implemented with 14 Finnish nurse prescribers using semi‐structured individual interviews. The data were analyzed with inductive content analysis.

**Findings:**

Altogether, this study identified four main categories for experiences on role transition. These main categories are in role transition during nurse prescribing education (1) learning decision‐making; (2) meaningfulness of studies during education and at the beginning of nurse prescriber role at work; (3) development of expertise; and (4) significant change in work.

**Discussion:**

The meaningfulness of the learning and learning decision‐making are important in role transition during nurse prescribing education. There is a significant development of expertise and change in work procedures during the transition from a nurse to a nurse prescriber.

**Conclusions and Implications for Nursing:**

Nurse prescriber education has a key role in the transition experience from registered nurse to nurse prescriber. It is also possible that healthcare units do not utilize nurse prescribers effectively.

## 1 Introduction

Nurse prescribing has increased worldwide, bringing a new importance for nursing. According to the International Council of Nurses (ICN), nurse prescription is in use in 45 countries. Fourteen European countries have nurses with prescription authority. Globally, nurse prescribing as an extended role of nurses has been seen as part of the role of nurse practitioners (NP) (ICN 2021). Nurse prescribers provide holistic and efficient care for patients (Hammarberg et al. 2024) so that they obtain access to care quickly and therefore the outcomes for patients have improved (ICN 2021; Laapio‐Rapi et al. 2019). However, there are differences in nurse prescribing education between countries. In Canada, nurse prescribing is a part of NP education, while in other countries, it is an additional education on a master's level, for example, in the UK and Finland (ICN 2020).

Finnish nurse prescribing education (45, the European credit transfer and accumulation system (ECTS) and European Qualifications Framework (EQF) 7) consists of learning to be independent in clinical decision‐making. The decision‐making is based on a comprehensive health and physical assessment of the patient, pharmacology, prescribing the medicine, pathology, legislation, and ethical questions. Decisions have a focus on the whole person and are based on evidence‐based guidelines (Finnish National Core Curriculum for nurse Prescriber Education 2023; ICN 2020).

## 2 Background

Role transition refers in this study to the nurse role transition from the registered nurse to the nurse prescriber. The extended role of nurse prescribing has enhanced the attractiveness of a nursing career and elevated both autonomy and professional status, which in turn contributes to an enhanced reputation of the profession (Haririan et al. 2022; Wit et al. 2024). The role expansion allows nurse prescribers to deliver more holistic and person‐centered care and therefore increase the efficiency of patient care (Hammarberg et al. 2024). Furthermore, the job satisfaction of nurses has increased, alongside the opportunities for enhanced collaboration with multi‐professional colleagues (Hammarberg et al. 2024; Wit et al. 2024).

In this research, role transition is defined according to Duchscher and Widney (2018) and described by the stages of transition‐by‐transition shock model. In this model, the scope is on physical, intellectual, emotional, developmental, and sociocultural changes. The focus is on relationships, roles, responsibilities, knowledge, and performance expectations of the person who is in role transition. Role transition can be divided into three stages: doing, being, and knowing. In the first phase, doing, nurses attend to learn and integrate the new work. In the second phase, nurses begin to feel comfortable in their new role and they start questioning the healthcare system. In the third phase, nurses reach professional identity and independence (Duchscher and Widney 2018).

In the role transition process, nurses critically analyze their own previous knowledge, conceptions, and basic assumptions in the light of new knowledge. This leads to the transformation of their knowledge and thinking. This process includes social process, problem orientation, and the complexity of problems (Liitos et al. 2012). Learners construct knowledge based on individual experiences, beliefs, and attitudes. In so doing, they are adding new knowledge to previous subjective knowledge (Rannikmäe et al. 2021).

The transition to the advanced nursing role can be complex, and stressful career change can be challenging personally and professionally and cause mixed emotions. Learning new competencies and new reasoning skills takes time (Barnes et al. 2021; MacLellan et al. 2015). Nurses have demonstrated positive attitudes, understanding, and motivation toward their emerging role as nurse prescribers (Fox et al. 2022; Haririan et al. 2022).

In the new role as a nurse prescriber, clinical decision‐making transitions to another level compared with working as a nurse, and this can be a complex process. Nurse prescribers use both independent and collaborative decision‐making. They facilitate and evaluate new knowledge and alternatives in their clinical autonomy (Levy‐Malmberg et al. 2024). They are capable of applying competencies in both familiar and new situations, having good teamwork, demonstrating creativity, possessing a high level of self‐efficacy, understanding how to learn, and identifying factors that influence the scope of practice (Hako et al. 2023). Learning new competencies and new reasoning skills takes time and role transition can be challenging when registered nurses move up to the next level in their competence (MacLellan et al. 2015). However, there is a lack of research concerning role transition during nurse prescriber education and working in a new role.

## 3 The Aim of the Study

The aim of this study was to describe nurse prescribers’ experiences of role transition during nurse prescriber education and the first working years in a new role. The goal of this study is to strengthen the knowledge and achieve an understanding of the nurse prescriber role transition. The research questions were: What experiences are associated with the transition from the registered nurse to the nurse prescriber during nurse prescriber education? What experiences do nurse prescribers have as regards role transition?

## 4 Methods

### 4.1 Study Design

This study is associated with phenomenology, which is reflected in the study's aim, the research questions and the lived experiences of nurses described about role transition during their prescribing education and also working in new roles (Gray 2021, van Manen 2017). This descriptive study was implemented by semi‐structured interviews.

### 4.2 Study Setting and Recruitment

The study setting involved semi‐structured interviews with participants from June 2022 to December 2023 to explore their experiences in the study aim by the principal researcher. The interviews were conducted with TEAMS connection, at a time that was convenient for the participants and in a friendly atmosphere.

Recruiting participants for interviews was purposive, providing in‐depth knowledge concerning the study questions (Gray 2021), and it was implemented in different ways. The recruitment notification was published through a nurse prescribers’ electronic newsletter published by a Finnish nurse prescribers’ association and their Facebook group. In the first recruitment notification, the inclusion criteria for participants were as follows: the participant had to be a registered nurse, have the right to prescribe medicines, and have been doing this work for one to two years. Eight interviewees were recruited in this round. After this, it was decided to extend the working experience to three years. Therefore, the recruitment notification was published for a second and third time to achieve more interviewees. Moreover, the researcher gave a short speech concerning research recruitment at a seminar for nurse prescribers. Interviewees and seminar participants were asked to circulate the information concerning research, after which a snowball effect was used (Gray 2021). After these actions, the researcher obtained six more interviews. There were 14 nurse prescribers enrolled in the study.

### 4.3 Data Collection

Data were collected by using individual, semi‐structured interviews online at Microsoft TEAMS (Gray 2021). Creation of the framework for interviews started with a literature review to identify the concepts and provide familiarization with ongoing research areas. After this, preliminary semi‐structured interview themes were formulated. These were discussed by two nursing science professors and some clarification was made. After pilot testing, the themes were found to be usable, and a pilot interview was included in the data (Kallio et al. 2016).

It was decided to take two nurse prescribers who had three years longer work experience than was necessary for the inclusion criteria into the research after the interviews were transcribed. This was because it was seen from transcriptions that their answers were similar to the others, and they increased the depth of information (Grove 2021).

The interviews started with two background questions and then proceeded to two other themes. The research questions were: What experiences are associated with the transition from registered nurse to nurse prescriber during nurse prescriber education? What experiences do nurse prescribers have on role transition?

These experiences concerned the elements that are associated with the development of decision‐making skills and the transition from registered nurse to nurse prescriber (Kotila et al. 2020, Kallio et al. 2016). The interviews took 30–60 minutes and were recorded and saved before transcribing. Interviews were transcribed into a text format (Gray 2021).

### 4.4 Ethical Considerations

The human sciences ethical committee of the University of Turku was applied for an ethical review statement (approval number 15/2022). Good scientific practice, research ethics, and participants’ rights were considered throughout the study process (TENK 2023). Participation was voluntary and written informed consent was obtained. Only the principal researcher had access to participants’ names and e‐mail addresses. Participants’ personal information was destroyed immediately after the interview, as promised in the recruitment process. Data were anonymized (ALLEA 2023). Transcriptions were anonymized and stored on a secured cloud server, with a password (ALLEA 2023; TENK 2023). During the entire process, the principal researcher was careful with trustworthiness, as indicated by credibility, dependability, and transferability in qualitative studies (Graneheim and Lundman 2004). The COREQ checklist was used to guide the reporting (Tong et al. 2007).

### 4.5 Data Analysis and Synthesis

Data were analyzed with inductive content analysis in February 2024. The principal researcher read the transcriptions several times to form an understanding of the data. (Graneheim et al. 2017). The analysis started with identifying meaning units that answered the aim of the study and research questions. In the next phase, synthesizing and coding were detailed, trying to keep codes on the same level as the original text. In this way, the risk was minimized as regards missing important content. In the third phase, codes were interpreted and sorted into subcategories (Lindgren et al. 2020). Next, similar content was searched for and identified in order to form categories. From these 10 categories, four main categories were formed, and from the main categories, two combining themes (Graneheim et al. 2017). NVivo and Exell were used to help in managing the data. (Graneheim and Lundman 2004; Graneheim et al. 2017).

## 5 Findings

**FIGURE 1:**
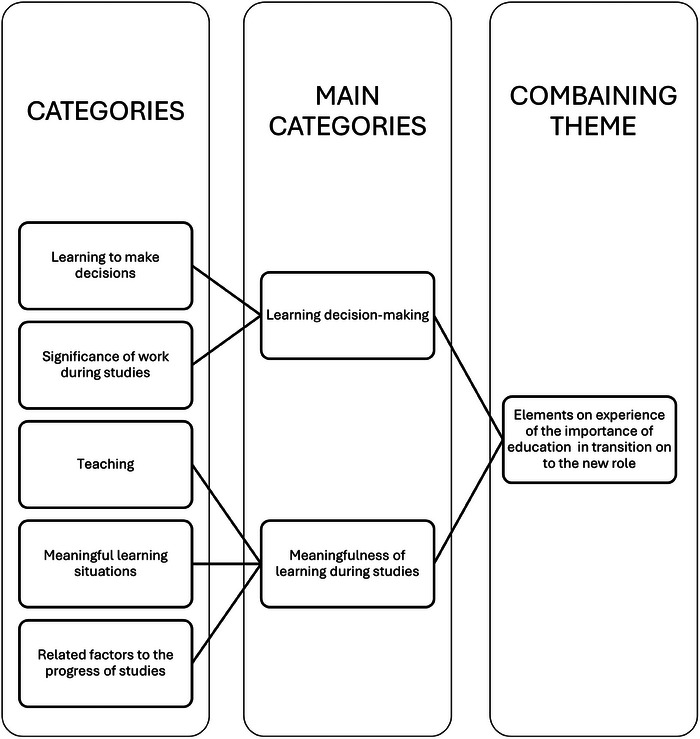
Experiences associated with the role transition during nurse prescribing education.

**FIGURE 2:**
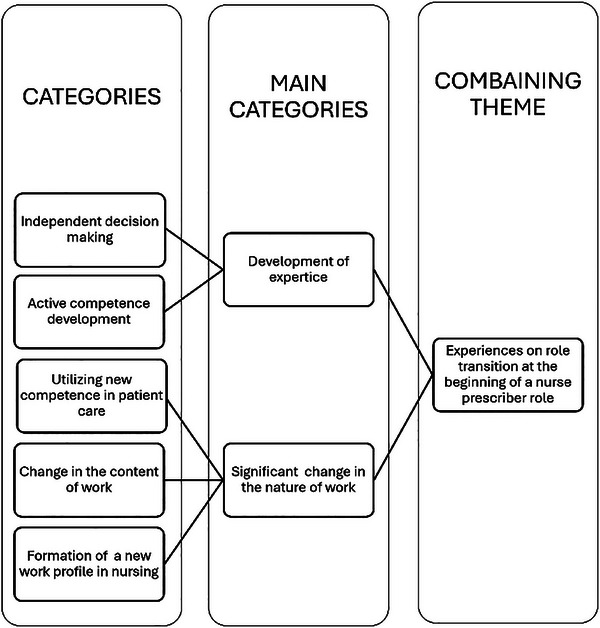
Nurse prescribers’ experiences on role transition.

Finnish nurse prescribers (*N* = 14) participated in this study. Participants were working in primary healthcare settings and had prescribing experience ranging from 12 months to six years. The working areas were from family planning to emergency units and outpatient clinics that treat patients with conditions such as asthma or diabetes. The experiences associated with the role transition from nurse to nurse prescriber during nurse prescribing education (Figure 1) were described as learning decision‐making and the meaningfulness of learning during studies. Nurse prescribers described their experiences of role transition (Figure 2) through experiences in the development of expertise and the significant change in the nature of their work.

### 5.1 The Experiences Associated With Role Transition During Nurse Prescribing Education

#### 5.1.1 Learning Decision‐Making

The experiences associated with learning decision‐making were learning to make decisions and the significance of work during studies. Learning to make decisions was described to be significant as meaningful learning situations and learning new, deepening of competence such as deeper understanding of the subject and learning skills; the development of decision‐making through developing knowhow, increasing self‐confidence and decision‐making and the meaning of the certification for self‐esteem.

The significance of work during studies was learning in the workplace, the significance of the working community, and increased responsibility in nursing. At the workplace, the support of the ward manager was important. Often the nurse prescribers experienced that the workplace managers had little knowledge about the education they had been given.

We've been incredibly fortunate that our supervisor has understood the importance of nurse prescribing education and this opportunity for nursing. (R3)

The significance of the working community during studies was described as the positive support of workplace colleagues and doctors. It was described as enthusiasm, endorsement, and positive comments. However, there were also negative views concerning nurse prescribing from doctors, like resistance and disbelief as regards the nurses’ new skills.

The doctors who work with us have given a lot of positive feedback on our professional skills. (R2)

Moreover, there was increased responsibility for nurses. The work of nurses had already changed during their nurse prescribing studies, and the nurses experienced that they grew into their new role during nurse prescribing education.

### 5.2 Meaningfulness of Learning During Education

The meaningfulness of learning included teaching, meaningful learning situations, and related factors to the progress of studies. Teaching was experienced through the structure of studies and actions of the educators.

Unwavering support from tutor teachers was crucial. (R2)

During education, it was experienced that learning took place through meaningful learning situations, the content of the studies, learning together, and patient‐centered learning situations. Examinations were meaningful, for example, the Objective Structured Clinical Examination (OSCEs), pharmacology examinations, and the final examinations.

The most memorable learning situations came from my failures…I thought I knew something, and then it turned out I didn't. (R1)

Related factors to the progress of studies were experienced through challenges during the studies such as the difficulty of subjects and controlling time consumption. Thus, the factors promoting studies were the students’ motivation for the studies, the support of companions during studies, and coping with studies.

Exams were difficult. (R4)

### 5.3 Nurse Prescribers’ Experiences on Role Transition

#### 5.3.1 Development of Expertise

The development of expertise was divided into two categories: independent decision‐making and active competence development. Independent decision‐making included growth in self‐confidence, belief in own actions, confidence, and expressed pride in themselves; they had a high increase in self‐esteem and developed more self‐confidence. Nurse prescribers expressed that they felt they had developed in their roles. However, they also felt a fear of mistakes. Independent decision‐making also included the development of decision‐making skills, such as understanding, justifying, and the development of independent decisions.

When knowledge is growing, grows self‐confidence. (R11)

The development of expertise can be seen as active competence development. Nurse prescribers experienced that they were active knowledge seekers. They were eager for new knowledge, pursuing knowledge, and educating themselves in their spare time.

You have to be motivated, curious and interest in things. (R1)

I use a lot of different databases in searching knowledge. (R13)

Active competence development was also described as learning new competencies. Often learning and education occurred in the workplace.

Physician on our workplace teaches us all the time. (R14)

### 5.4 Significant Change in the Nature of Work

The significant change in the nature of work included three categories: utilizing new competence in patient care, change in the content of work, and formation of new work profile in nursing. Utilizing new competencies in patient care includes deep understanding and administration of medication management, usage of new competencies in the assessment of the need for treatment, and identifying the need for patient education. These competencies were clinical examination of patients, differential diagnostics, and assessment of the need for treatment during an appointment or on the phone. Nurse prescribers experienced that their capabilities of assessing the patient's situation and giving advice to patients over the phone were better. They experienced a deeper understanding and implementation of medication management and how their knowledge concerning medication grew. They learned to prescribe, and they paid more attention to medication management. Utilizing new skills in assessing the need for treatment consists of working on the phone, listening to the patients more intensely, developing patient education, clinical examinations of the patient, widening the patient material and differing diagnostics development.

If I were regular nurse, I could not do clinical examination for patients as I can now. (R5)

The change in the content of work includes career advancement, making work visible, change in the nature of work, and knowledge sharing. Nurse prescribers experienced more freedom in their work. They had the opportunity to create their new work description, had more responsibility, and took care independently, for example, of acute care like infections, and chronic diseases like asthma and diabetes or blood pressure controls. They felt their career was advancing and making their work visible, significant, and becoming meaningful as nurse prescribers.

This is one opportunity for career advancement… and to become more visible at work. (R3)

Work was more independent, the responsibility was greater than before, and patient groups changed. Nevertheless, there were also challenges in this change, like sleepless nights, anxiety, and fear. Challenges were faced by the human resources department and many nurse prescribers had to create their own work description. Nurse prescribers also spoke about knowledge sharing. They taught and shared a large amount of knowledge not only with their colleagues but also outside of their working places, for example, in nursing education.

The formation of new work profiles for nurses was seen in colleagues’ attitudes toward their new skills and work arrangements within a work unit. Colleague's attitudes varied. They could be supportive and trusting but also suspicious. Nurses who did not know much about nurse prescribing had negative attitudes toward the new roles, as did many doctors. However, as knowledge concerning the role of nurse prescribers in the workplace rises, the more attitudes will change to being positive.

One senior physician viewed statistics and found out patients who were taken care by nurse prescribers got to the appointment quickly, got their examination and treatment quickly and went back home quickly. (R1)

Work arrangements in the workplaces were crucial. The supervisors’ role was important. Thus, there were differences in how new work descriptions were enabled in workplaces. Many nurse prescribers experienced that their competence was not used in the proper way and felt that they could give much more expertise to the patients’ care and to the work of the unit.

I felt that I had to remind all of the time that please give me these patients which I can take care. (R6)

## 6 Study Limitations and Strengths

This study has some limitations, which are related to the sample and to the principal researcher. This study aimed to recruit participants whose experience met the inclusion criteria. The data collection used both direct recruitment in newsletters directed to Finnish nurse prescribers, and in their Facebook group, a snowballing method was also employed. It is possible that these recruitment methods did not reach all nurse prescribers who would have been interested in participating in the research (Grove 2021). However, informative, deep, and rich data were obtained. As a member of the Finnish Higher Education Network of Nurse prescriber Education, there is a potential for bias due to the principal researcher's close relationship with nurse prescribing education and its national development. However, this can also be seen as a strength, and efforts were made to approach the data with an open mind. The strengths of this study were the framework construction of the interviews and utilizing online interviews. The framework for the semi‐structured interviews was conducted by following the guidelines for qualitative semi‐structured interviews (Kallio et al. 2016). Online interviews offered flexibility to both the researcher and participants (Gray 2021).

## 7 Discussion

The findings describe nurse prescribers’ experiences of role transition during nurse prescriber education and at the beginning of their work as nurse prescribers. Understanding the role transition from registered nurse to nurse prescriber is important both from the viewpoint of education and implementing the nurse prescriber role into practice. Nurse prescribers described their experiences of transition to new roles comprehensively and in detail. With inductive content, the analysis defined two main themes as regards how nurse prescribers described the role transition to the new role: these themes were experiences associated with the role of transition during nurse prescribing education and nurse prescribers’ experiences of role transition.

The experiences associated with role transition during nurse prescribing studies were learning decision‐making and the meaningfulness of learning during education. The importance of education in the transition to a new role was described concretely by nurse prescribers. They described different kinds of learning situations, which were important during education, for example, learning together and OSCE exams. Nurse prescribers described that during education, it was important to talk and compare different nursing practices and encourage each other. OSCE exams were described as being difficult but educational. In these exams, nurse prescribers realized that they are really stepping into a new role, in which there are new skills that they have to develop and they have to make decisions concerning patients’ treatment. Similar findings were found in a study by Montgomery et al. (2021), where the OSCEs were seen to be not only useful to learning and education as a tool for clinical competence but also beneficial in the improvement of clinical competence and knowledge. However, OSCEs were also seen as challenging, as they caused stress and anxiety, but were not found to be intimidating (Montgomery et al. 2021).

There were also thoughts concerning competencies before nurse prescriber education. Students have at least three years of work experience, but many students have much more, and they are very capable and competent registered nurses. It is not easy to be a student again and notice that there is a lack of knowledge concerning the new nursing area. The work experience before starting nurse prescribing education is important for nurse prescriber students. This way, they understand nursing phenomena; they recognize the roles between physicians and registered nurses, and they can create and grow to a new role as a nurse prescriber: a role that is between nurses and physicians. Nurse prescribing education takes about one year (45 ECTS) depending on the students and their situation. With these study findings, it can be seen that nurse prescriber students go through role transition during one year of nurse prescribing education (Duchscher and Widney 2018), but the new role transition, to some extent, starts again when working in the new role.

Nurse prescribers’ experiences of role transition consist of the development of expertise and significant change in the nature of their work. Nurse prescribers experienced that they are competent in many ways. They described how their competence has increased; they can perform clinical examinations of patients, and they understand what the findings mean, and based on these, they make their conclusions and decisions concerning the patient's health. They have been listening to lungs and checked ears before as registered nurses, but after nurse prescriber education they have extended their knowledge, and they can make differential diagnostics. Nurse prescribers described how these competencies help them when working in emergencies, at appointments, and on the phone; in all these areas, they are more confident. Nurse prescribers also recognize when they must send patients for a physician's evaluation. Nurse prescribers described their motivation and understanding toward more demanding duties in their new role. Nurse prescribing education seems to provide a good foundation for the role transition from nurse to nurse prescriber. These findings can be seen in studies by Fox et al. (2022) concerning improved patient healthcare experience; by Hammarberg et al. (2024) regarding holistic, effective, and patient‐centered care; and by Wit et al. (2024) regarding the positive impact on professional autonomy.

There is still resistance from physicians to nurse prescribing. Nurse prescribers described situations where some physicians did not believe in their new skills or thought that nurse prescribers would take some easier patients from them as Wit et al. (2024) also found in the Netherlands. We need both professions to manage our patients and health care. Fortunately, in the study by Hammarberg et al. (2024), most nurse prescribers have supportive colleagues in physicians, managers, and nurses. In this study, it was found that nurse prescribers had at least one supportive colleague, either a physician, a nurse manager, or a colleague.

There is still much to do in implementing the nurse prescriber role into the workplace. At healthcare centers and workplaces, very often these new competencies of nurse prescribers are not recognized, which is why they might be underutilized. Nurse prescribers see their work with new skills could be utilized in many ways. Hence, this can be the reason nurse prescribers in this study suggested that it would be important for healthcare workers and the staff in workplaces to familiarize themselves with nurse prescriber education.

When these findings were compared with the transition‐by‐transition shock model, nurse prescribers experienced intellectual, emotional, developmental, and sociocultural changes, but not physical changes. There were also changes in relationships, roles, and responsibilities with colleagues, managers, and physicians. Nurse prescribers also experienced changes in knowledge and performance expectations. Considering the three stages (doing, being, and knowing) of role transition, there can be seen experiences with learning and integrating new competencies into the new work (doing). Nurse prescribers’ feelings concerning their new role varied between comfortable and uncomfortable, and there were questions about how the healthcare system utilizes nurse prescribers (being). They attained a new professional identity and partial independence depending on how workplaces organized the work of the nurse prescribers (knowing) (Duchscher and Widney 2018).

## 8 Conclusion

Nurse prescribers experienced role transition by increasing and extending their competence in their work. They describe themselves as highly motivated, but they recognized their competence boundaries. According to this study, it is possible that healthcare units do not recognize the new competencies nurse prescribers have, and that is why they are underutilized. Nurse prescribing education is an important part of the role transition experience from registered nurse to nurse prescriber. It is not easy for experienced nurses to note that there is a lack of knowledge concerning the new nursing area. The meaningfulness of learning and learning decision‐making are important in this process. With this knowledge, it is possible to develop nurse prescriber education and competencies, and in this way, we can improve patient safety and the quality of nurse prescribing. It could be beneficial to research the transition in healthcare units.

## 9 Implications

This research provides a new perspective on the role transition from the nurse to the nurse prescriber. During nurse prescriber education, learning decision‐making and meaningfulness of learning are important issues. It is important to use comprehensive learning methods in nurse‐prescribing education to help nurses transition into nurse prescribers. It is crucial to deliver information concerning nurse prescribers’ competencies to healthcare units, to doctors, and to nursing managers. With this knowledge, the experience of nurse prescribers in the workplace could be enhanced during their education and their new role as nurse prescribers could be utilized effectively. During the first working years in a new role, the development of expertise and significant change in the nature of work are important issues. These findings also describe the experiences of nurse prescribers’ development in clinical competencies and might help in the implementation of this new role in practice. The findings can be used in developing strategies for nurse prescriber education and the implementation of nurse prescriber roles in healthcare practices.

## Author Contributions

Study design: JS, LS, and HV. Data collection: JS. Data analysis: JS, LS, and HV. Study supervision: LS and HV. Manuscript writing: JS, HV, VS, and LS. Critical revisions for important intellectual content: LS, MR, VS, and HV. All authors approved the final version for submission.

## Conflicts of Interest

The authors declare no conflicts of interest.
